# Combined Pericapsular Nerve Group (PENG) and Ilioinguinal-Iliohypogastric Blocks for Pediatric Pelvic Osteotomy: A Tailored Regional Anesthesia Approach

**DOI:** 10.7759/cureus.103768

**Published:** 2026-02-17

**Authors:** Rayan Muawad, Abdullah AlDhuwaihy, Khalid Alanezi, Faisal Alshuwaier, Mohammed M AlRuwaili, Nawaf Faisal S Alsubaie, Mostafa Nagy

**Affiliations:** 1 Pediatric Anesthesia, King Abdullah Specialist Children Hospital, Ministry of National Guard Health Affairs (MNGHA), Riyadh, SAU; 2 College of Medicine, King Saud University, Riyadh, SAU; 3 College of Medicine, Majmaah University, Al-Majma'ah, SAU

**Keywords:** developmental dysplasia hip (ddh), iliohypogastric-ilioinguinal nerve block, pediatric-anesthesia, pediatric regional anesthesia, pericapsular nerve block (peng), ultrasound guided nerve block

## Abstract

The pericapsular nerve group (PENG) block is a regional anesthesia technique that targets the articular branches innervating the anterior hip capsule, offering effective analgesia with minimal motor blockade. While its utility in hip surgery is established, its solitary use in pediatric pelvic osteotomies leaves the cutaneous incision site uncovered. We report the case of a 21-month-old male with significant cardiopulmonary comorbidities undergoing a unilateral pelvic osteotomy. To minimize respiratory and hemodynamic risks associated with opioids, a multimodal, opioid-sparing analgesic strategy was implemented. This approach combined an ultrasound-guided PENG block with an ipsilateral ilioinguinal-iliohypogastric nerve block to ensure comprehensive coverage of both deep articular and superficial somatic pain. The intraoperative course was stable, and the patient demonstrated excellent postoperative analgesia with no requirement for rescue opioids. This case highlights that supplementing the PENG block with an ilioinguinal-iliohypogastric block provides a tailored, anatomical solution for pediatric pelvic osteotomy, offering a safe and effective regional anesthesia strategy for high-risk pediatric patients.

## Introduction

Pediatric orthopedic surgical procedures require a comprehensive approach to perioperative pain management that prioritizes patient comfort and safety [1]. Regional anesthesia is increasingly favored over local infiltrative anesthesia (LIA) in these settings because it can offer broader anatomical coverage and facilitate opioid-sparing strategies [1,2]. Recent studies suggest that peripheral nerve blocks may contribute to improved pain control and enhanced early recovery in orthopedic patients [3,4].

Common regional techniques for hip surgery, such as the fascia iliaca compartment block (FICB) or femoral nerve block (FNB), are effective but are frequently associated with quadriceps muscle weakness, which can delay mobilization [5].

The pericapsular nerve group (PENG) block, first described in 2018 by Girón-Arango et al., was developed to overcome this limitation [2]. By selectively targeting the articular branches of the femoral, obturator, and accessory obturator nerves, the PENG block aims to provide hip analgesia while minimizing motor blockade [2,6].

However, the application of the PENG block in pediatric pelvic osteotomies presents a unique challenge: the standard PENG block does not cover the cutaneous dermatomes of the anterior abdominal wall and groin, which are incised during this procedure. We present the case of a 21-month-old patient with an atrial septal defect (ASD) and recurrent bronchitis, where a PENG block was combined with an ilioinguinal-iliohypogastric nerve block (ILIHB). This tailored approach aimed to provide complete analgesic coverage while minimizing perioperative respiratory and cardiovascular risks.

## Case presentation

Case presentation

A 21-month-old male weighing 14 kg presented for a left pelvic osteotomy to treat developmental dysplasia of the left hip (DDH). His medical history included a secundum atrial septal defect (ASD II) and recurrent bronchitis, with a history of repeated hospital admissions for chest infections. Routine preoperative laboratory investigations were within normal limits. Given the history of ASD, preoperative echocardiography was obtained to assess cardiac status, revealing normal ventricular function with a left-to-right shunt and no pulmonary hypertension.

Standard American Society of Anesthesiologists (ASA) monitoring was applied. Anesthesia was induced with sevoflurane in oxygen, followed by propofol (2 mg/kg). To minimize airway irritation and the risk of perioperative bronchospasm, a significant concern given the patient’s history of recurrent bronchitis, a laryngeal mask airway was selected for airway management. Anesthesia was maintained with sevoflurane in an air-oxygen mixture, and intravenous acetaminophen (15 mg/kg) was administered intraoperatively to initiate the multimodal, opioid-sparing analgesic protocol.

Regional anesthesia technique

**Figure 1:**
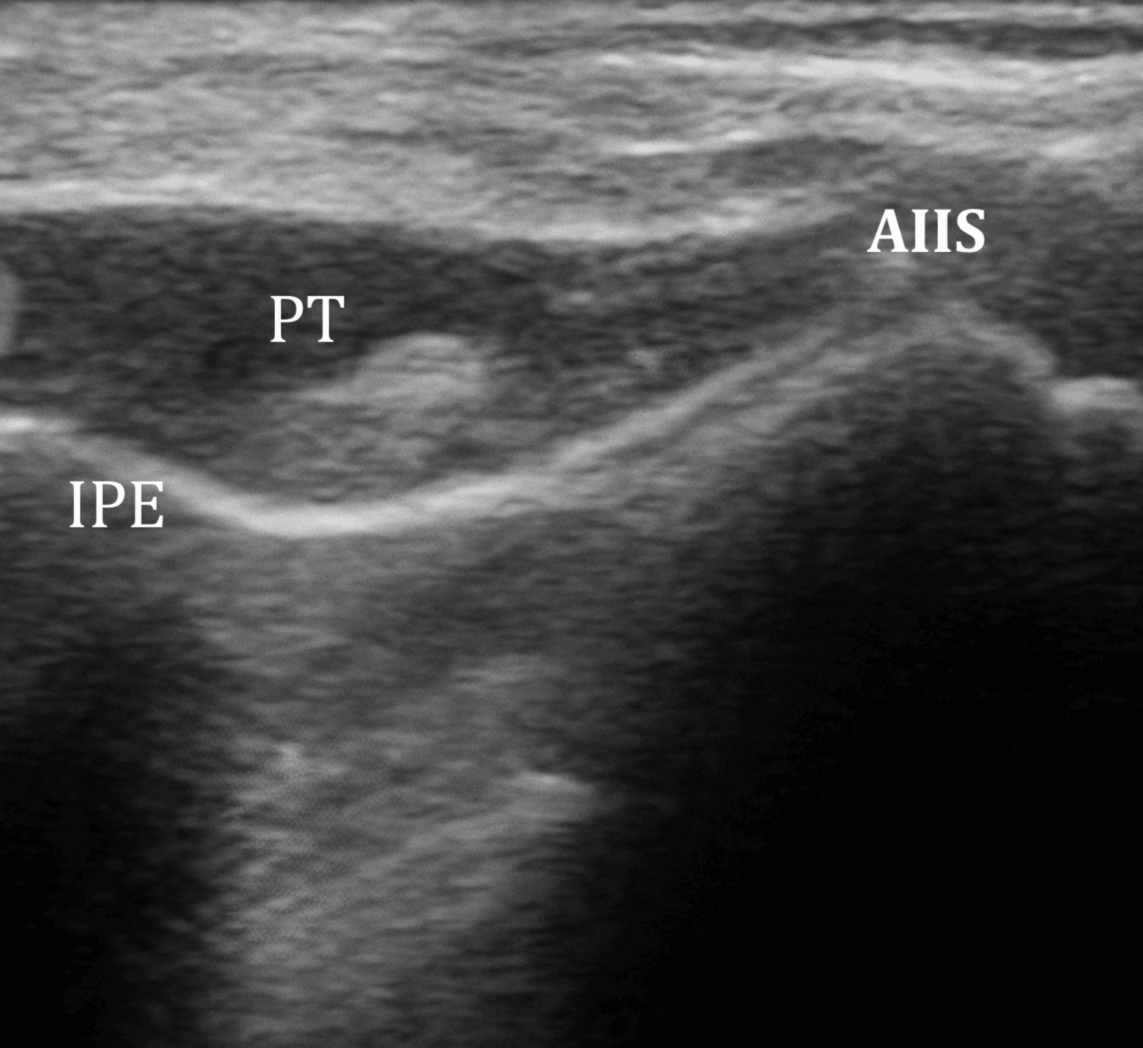
Ultrasound-guided PENG block landmarks and injectate deposition. The image displays the relationship between the anterior inferior iliac spine (AIIS), the psoas tendon (PT), and the iliopubic eminence (IPE). The local anesthetic is visualized as a hypo-echoic (dark) expansion in the fascial plane directly beneath the PT and superficial to the pubic ramus. FA, femoral artery; PENG, pericapsular nerve group

Under real-time ultrasound guidance and aseptic technique, a high-frequency linear probe was positioned over the anterior inferior iliac spine (AIIS) and aligned with the pubic ramus to visualize the iliopubic eminence (IPE), psoas tendon (PT), and femoral artery (Figure 1).

An echogenic needle was advanced in-plane toward the fascial plane between the PT and the pubic ramus. Following negative aspiration, a PENG block was performed using 7 mL of 0.25% bupivacaine, with the injectate spread visualized beneath the PT.

To address the cutaneous innervation of the surgical incision, which involves dermatomes not covered by the PENG block, an ipsilateral ultrasound-guided ILIHB was subsequently performed using 4 mL of 0.25% bupivacaine. The addition of this block was justified by the location of the surgical incision; in pediatric open DDH surgery, the approach is typically anterior and positioned close to the groin, rather than the lateral approach often favored in adult hip procedures. The total dose of bupivacaine administered was 27.5 mg (1.96 mg/kg), which remained well below the maximum recommended limit of 3 mg/kg.

The anterior surgical approach for pelvic osteotomy involves an incision primarily within the L1 and L2 dermatomes. Since the PENG block targets only the deep articular branches to the hip capsule (femoral, obturator, and accessory obturator nerves), the addition of the ilioinguinal-iliohypogastric block was essential to provide complete cutaneous anesthesia for the groin-based surgical site.

Intraoperative and postoperative course

The surgical procedure proceeded uneventfully with stable hemodynamics, and no intraoperative rescue opioids were required. Postoperatively, the patient exhibited excellent pain control, with Face, Legs, Activity, Cry, Consolability (FLACC) scale scores remaining consistently below 2 for the first 12 hours. Scheduled intravenous acetaminophen (15 mg/kg every six hours) was administered as part of the multimodal analgesic protocol, with the first postoperative dose given six hours after the intraoperative dose. The patient required no rescue opioids throughout the hospital stay and was safely discharged 36 hours postoperatively. No complications, such as motor weakness or signs of local anesthetic systemic toxicity (LAST), were observed.

## Discussion

The PENG block is a regional anesthesia technique that has gained attention for providing analgesia for hip and pelvic surgeries. By targeting the articular branches of the femoral, obturator, and accessory obturator nerves near the iliopubic eminence, the PENG block aims to anesthetize the anterior hip capsule. This motor-sparing feature is particularly desirable in pediatric patients to facilitate early mobilization [1,2]. While the FNB is a traditional choice for hip surgery, it is frequently associated with quadriceps weakness, which can complicate early assessment. The PENG block may offer potential advantages by targeting only the sensory articular branches, potentially preserving motor function in the immediate postoperative period [5,6].

Regional anesthesia techniques are a cornerstone of perioperative analgesia in pediatric anesthesia and have been associated with improved pain control and reduced opioid consumption [3,4]. The PENG block was first described in 2018 by Girón-Arango et al. [2]. Since its introduction, evidence has accumulated regarding its utility in adult hip surgery, and more recently, its application in pediatric populations [7,8]. In the present case, the decision to combine the PENG block with an ILIHB was guided by the location of the surgical incision [9,10]. Pediatric pelvic osteotomy is typically performed through an anterior incision close to the groin. While the PENG block targets deep articular structures, it does not reliably cover the superficial tissues of the lower abdominal wall and groin [11].

While much of the foundational evidence for the PENG block’s mechanism originates from adult orthopedic literature [2,5], the anatomical landmarks - specifically the relationship between the PT and the hip capsule - remain consistent and applicable in the pediatric population [12,13]. Weight-based dosing remains a critical consideration in pediatric regional anesthesia to minimize the risk of LAST. In our experience and based on emerging small case series, the PENG block demonstrates a favorable safety profile; however, meticulous weight-based dosing remains mandatory to avoid complications.

To our knowledge, there are few published reports describing the combined use of the PENG block and ILIHB in pediatric pelvic osteotomy. This technique appeared to provide effective multimodal analgesia in this clinical scenario, evidenced by the patient’s low postoperative FLACC scores and the complete absence of rescue opioid requirements. Compared with caudal or neuraxial analgesia, this peripheral approach may offer advantages regarding unilateral blockade and reduced risk of urinary retention [11]. Overall, this case supports the exploration of the PENG block as a component of multimodal analgesia for pediatric hip surgery and suggests a potential benefit in supplementing it with ILIHB for anterior pelvic incisions [12,14].

Limitations

While the successful outcome in this case highlights the potential of the PENG and ILIHB combination, the inherent limitations of a single case report must be acknowledged. The findings cannot be generalized to all pediatric patients, and the safety profile, particularly regarding the risk of LAST in infants, requires further investigation. Prospective, randomized controlled trials are necessary to establish the definitive efficacy and safety of this regional technique compared to traditional methods in pediatric orthopedic surgery.

## Conclusions

In conclusion, the combined PENG and ILIHB provided a tailored analgesic approach for this pediatric patient undergoing pelvic osteotomy. In this clinical instance, the technique was associated with effective postoperative pain control and a lack of rescue opioid requirements, while no postoperative motor weakness was observed. This case demonstrates the potential clinical utility of this regional combination, particularly for patients with complex comorbidities. However, these observational findings should not be generalized, and further prospective, randomized studies are necessary to definitively establish the safety and efficacy of this approach in the broader pediatric population.
